# Global awareness and integration of immunotherapy among thoracic surgeons: a multicenter survey study

**DOI:** 10.1590/1806-9282.20250540

**Published:** 2026-01-09

**Authors:** Muharrem Özkaya, Nilay Cavusoglu Yalcin, Ayşegül Güler, Arif Hakan Önder, Halil Göksel Güzel

**Affiliations:** 1 University of Health Sciences, Antalya Training and Research Hospital, Department of Thoracic Surgery – Antalya, Turkey.; 2 University of Health Sciences, Antalya Training and Research Hospital, Department of Medical Oncology – Antalya, Turkey.

**Keywords:** Lung neoplasms, Thoracic surgery, Immunotherapy

## Abstract

**OBJECTIVE:**

Lung cancer is the leading cause of cancer-related mortality, with non-small cell lung cancer accounting for most cases. Surgery remains the primary treatment for early-stage non-small cell lung cancer, while advanced disease requires multimodal strategies. Immunotherapy, particularly immune checkpoint inhibitors targeting programmed cell death protein-1, programmed death-ligand 1, and cytotoxic T-lymphocyte-associated protein 4, has significantly improved survival and is increasingly incorporated into neoadjuvant and adjuvant settings. The aim of the study was to evaluate thoracic surgeons’ awareness, perceptions, and integration of immunotherapy in surgical practice.

**METHODS:**

A cross-sectional survey was conducted among 64 thoracic surgeons from 19 countries to assess their perspectives, challenges, and regional disparities in immunotherapy adoption. Statistical analyses examined associations between perceptions and variables such as specialty, experience, hospital type, and geographic region.

**RESULTS:**

Perceptions of immunotherapy did not differ significantly by specialty, experience, hospital type, or geographic location. However, significant associations were observed between years of experience and hospital type, as well as surgical specialty and geographic region. Key barriers included cost, lack of standardized guidelines, and challenges in multidisciplinary coordination. Surgeons in low- and middle-income countries reported greater difficulties in drug access and institutional infrastructure. The majority of participants (85%) expressed a need for further training, highlighting the importance of structured education programs and enhanced collaboration between surgical and medical oncology teams.

**CONCLUSION:**

While immunotherapy is widely accepted among thoracic surgeons, challenges related to education, accessibility, and implementation persist. Addressing these barriers through global initiatives, cost-effective policies, and multidisciplinary cooperation is essential for optimizing immunotherapy integration in thoracic surgery and improving outcomes for non-small cell lung cancer patients.

## INTRODUCTION

Lung cancer remains the leading cause of cancer-related mortality worldwide, accounting for approximately 1.8 million deaths annually, with non-small cell lung cancer (NSCLC) constituting the majority of cases (80–85%)^1^. Surgery remains the cornerstone of treatment for early-stage NSCLC, providing the best chance for long-term survival^2,3^. However, many patients are diagnosed at an advanced stage of disease, where surgical resection alone is insufficient.

Recent advancements in systemic therapies, particularly immunotherapy, have significantly altered the therapeutic landscape of NSCLC^4,5^. Immune checkpoint inhibitors (ICIs), which target regulatory molecules such as programmed cell death protein-1, programmed death-ligand 1, and cytotoxic T-lymphocyte-associated protein 4, have demonstrated substantial improvements in progression-free survival (PFS) and overall survival (OS) outcomes in patients with advanced-stage NSCLC^6^. Furthermore, the incorporation of immunotherapy into neoadjuvant and adjuvant treatment settings has emerged as a pivotal strategy to optimize surgical outcomes by reducing tumor burden, enhancing immune-mediated tumor control, and improving long-term disease-free survival^7^.

Despite the growing evidence supporting immunotherapy, its successful incorporation into thoracic surgical practice still faces several challenges, including limited awareness among surgeons, educational gaps, and barriers to multidisciplinary collaboration^8^. Additionally, regional disparities in access to immunotherapy, institutional infrastructure limitations, and cost considerations contribute to variations in its implementation^9^.

This study evaluates thoracic surgeons’ awareness, perceptions, and opinions regarding the integration of immunotherapy into surgical treatment. By analyzing responses from surgeons across multiple regions, the study aims to identify key challenges, educational needs, and potential strategies for optimizing the use of immunotherapy in thoracic surgical practice.

## METHODS

### Study design and participants

This study was designed as a multicenter, cross-sectional survey, including 64 thoracic surgeons from 19 countries. Participants represented diverse professional backgrounds, including variations in experience level, institutional affiliation, and surgical case volume related to NSCLC. Participants were primarily invited through a global WhatsApp group of thoracic surgeons established by Dr. Diego González Rivas, the pioneer of uniportal video-assisted thoracic surgery. This international group, which connects practicing thoracic surgeons worldwide, served as the main platform for survey dissemination. Additional invitations were extended via professional academic networks and direct e-mail contacts to ensure wide participation.

### Data collection

The survey consisted of 19 multiple-choice and open-ended questions, administered via an online platform (SurveyMonkey) between December 1 and December 31, 2024. The questionnaire was organized into four thematic domains: (1) awareness and knowledge of immunotherapy, (2) perceptions and attitudes regarding its effectiveness and safety, (3) barriers and challenges to clinical integration, and (4) educational and training needs of thoracic surgeons. Prior to dissemination, the questionnaire was pilot-tested with three thoracic surgeons to ensure clarity, face validity, and ease of completion. Feedback from this pilot phase was used to refine the wording of selected questions.

### Statistical analysis

Statistical analyses were performed using IBM SPSS Statistics (version 28). Descriptive statistics were calculated for categorical variables as frequencies and percentages (%), while continuous variables were summarized as means and standard deviations. Non-parametric tests were selected because the survey responses were primarily ordinal in nature and the data distribution did not meet the assumptions of normality. Missing responses, which accounted for less than 5% of all items, were excluded listwise from the relevant analyses to maintain data integrity.

For group comparisons, non-parametric tests were used due to the ordinal nature of survey responses. The Kruskal-Wallis test was applied to assess differences in immunotherapy perceptions based on surgical specialty, experience, and hospital type. The Mann-Whitney U test was used to compare responses between academic and non-academic hospitals. Chi-square (χ²) tests were performed to evaluate associations between categorical variables, including experience level, geographic region, and hospital type. When necessary, Monte Carlo simulations were applied to confirm statistical robustness. A Pearson correlation analysis was conducted to assess the relationship between formal immunotherapy training and awareness (r=0.37, moderate positive correlation, p<0.05). All statistical tests were two-tailed, and a p<0.05 was considered statistically significant.

## RESULTS

**Figure 1:**
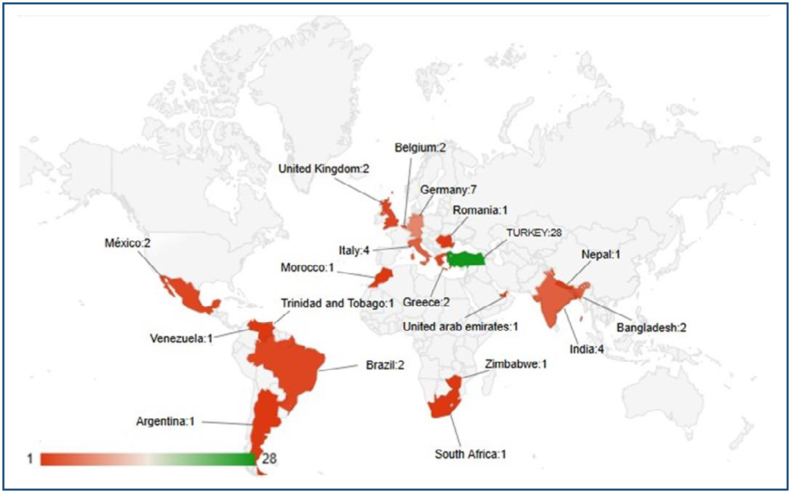
Global distribution of thoracic surgeons participating in the survey.

A total of 64 thoracic surgeons from 19 different countries participated in this study (Figure 1).

The statistical analyses provided the following findings:

A Kruskal-Wallis test was performed to assess differences in the perceived impact of immunotherapy among thoracic surgeons specializing in General Thoracic Surgery, Cardiothoracic Surgery, and other specialties. The results indicated no statistically significant differences (p=0.210), suggesting that perceptions regarding immunotherapy remain consistent across different surgical specialties.

The association between years of surgical experience (0–5, 6–10, 11–20, >20 years) and the perceived effectiveness of immunotherapy was analyzed using the Kruskal-Wallis test. No statistically significant association was found (p=0.644), indicating that clinical experience does not substantially influence surgeons’ perceptions of immunotherapy.

A Mann-Whitney U test was conducted to compare perceptions of immunotherapy between surgeons working in academic/university hospitals and those in non-academic institutions (private, public, or other hospitals). The analysis revealed no statistically significant difference (p=0.548), indicating that institutional affiliation does not affect surgeons’ attitudes toward immunotherapy.

A Kruskal-Wallis test was employed to evaluate whether geographic location influenced surgeons’ perceptions of immunotherapy. The analysis revealed no statistically significant regional differences among surgeons practicing in Europe, South America, Africa, and Asia (p=0.185), suggesting that opinions on immunotherapy remain consistent across different geographic regions.

A chi-square test was performed to assess the relationship between the continent of practice and hospital type. The results showed no statistically significant association (p=0.469). Monte Carlo simulation confirmed the robustness of this finding, reinforcing that hospital type is not significantly influenced by geographic location.

A chi-square test identified a statistically significant association between years of surgical experience and hospital type (p=0.018). However, linear-by-linear association testing (p=0.226) suggested no progressive trend. Cross-tabulation analysis further indicated that surgeons with 0–5 years of experience were primarily employed in academic hospitals, while those with 6–10 years predominantly worked in public hospitals, followed by academic hospitals. Surgeons with 11–20 years of experience were mainly affiliated with academic hospitals, whereas those with >20 years were mostly working in academic and private hospitals, with no representation in public hospitals.

A chi-square test was used to assess the distribution of thoracic surgical specialties across geographic regions, yielding a statistically significant association (p=0.001). Monte Carlo simulation confirmed the validity of this finding. However, linear-by-linear association testing (p=0.060) indicated no direct linear trend. Cross-tabulation analysis demonstrated that general thoracic surgeons were predominantly based in Europe, cardiothoracic surgeons were mainly located in Europe and Africa, with no representation in South America or Asia, and other surgical specialties had minimal representation, primarily in South America and Asia. Europe was the most represented geographic region overall.

A Mann-Whitney U test was conducted to evaluate differences in perceived immunotherapy impact between surgeons working in academic versus private hospitals. The analysis found no statistically significant difference (p=0.970), indicating that perceptions of immunotherapy are consistent across these hospital settings.

A Kruskal-Wallis test was conducted to assess whether years of surgical experience influenced perceptions of immunotherapy. No statistically significant association was found (p=0.644), suggesting that perceptions remain stable across different experience levels.

A Kruskal-Wallis test was employed to determine whether hospital type (academic, private, public, or other) influenced surgeons’ perceptions of immunotherapy. The analysis revealed no statistically significant differences (p=0.100), indicating that hospital type does not significantly impact perceptions of immunotherapy.

A Kruskal-Wallis test was used to evaluate whether geographic region influenced perceptions of immunotherapy. The results demonstrated no statistically significant differences (p=0.185), suggesting that geographical location does not substantially affect surgeons’ views on immunotherapy.

## DISCUSSION

The findings of this study highlight the growing acceptance of immunotherapy among thoracic surgeons worldwide as an effective adjunct to surgical treatment for NSCLC. A majority of participants perceived immunotherapy as "very effective" or "effective," aligning with evidence from clinical trials demonstrating its efficacy in improving PFS and OS^10^. This positive perception underscores the integration of immunotherapy into the surgical paradigm as a significant advancement in NSCLC management.

Despite this optimism, several barriers to implementation persist. Cost was the most frequently reported barrier, reflecting the substantial financial burden associated with ICIs^11^. Additionally, a lack of clear clinical guidelines and challenges in multidisciplinary collaboration were identified as significant obstacles. These findings are consistent with prior studies highlighting the need for standardized protocols and better coordination between surgical, medical oncology, and pathology teams to optimize patient outcomes^12^.

This study emphasizes the need for comprehensive training programs to prepare thoracic surgeons for the effective incorporation of immunotherapy into clinical practice. Given the rapid advancements in immuno-oncology, continuing medical education and targeted workshops should be prioritized to ensure that surgeons remain well-informed about emerging evidence, patient selection criteria, and optimal treatment sequencing. Furthermore, multidisciplinary collaboration between thoracic surgeons, medical oncologists, and radiation oncologists is critical to developing evidence-based guidelines that facilitate the seamless incorporation of immunotherapy into surgical workflows.

Educational deficiencies emerged as a critical area requiring attention, with 85% of participants expressing a need for additional training or workshops. This highlights the importance of ensuring thoracic surgeons have a comprehensive understanding of immunotherapy mechanisms, clinical indications, and perioperative considerations. Previous studies have demonstrated that focused educational initiatives can significantly facilitate the adoption of novel therapies and improve multidisciplinary decision-making^13^.

The findings also highlight the potential impact of regional disparities on the implementation of immunotherapy. Geographical disparities in immunotherapy access and practice patterns were also evident in this study. Participants from low- and middle-income countries reported greater challenges related to drug availability and institutional infrastructure. These observations are consistent with global analyses indicating that socioeconomic and healthcare infrastructure disparities profoundly affect equitable implementation of advanced cancer therapies^14^. Addressing these disparities requires global initiatives aimed at improving equitable access to immunotherapy, biomarker testing, and clinical trials, particularly in low- and middle-income countries, where healthcare resources are limited. Policies that promote cost-effectiveness and reimbursement strategies for immunotherapy should be explored to ensure broader implementation.

Additionally, the observed association between surgical specialty and geographic region suggests that practice patterns and institutional protocols may differ across regions, necessitating further investigation into how institutional frameworks and national healthcare policies shape surgeons’ perspectives on immunotherapy. Future research should explore whether specific institutional protocols or country-specific healthcare regulations influence the degree to which immunotherapy is integrated into thoracic surgery.

Overall, while immunotherapy has been widely accepted as a transformative advancement in the treatment of NSCLC, this study underscores the need for continued research, structured education, and international cooperation to optimize its integration into thoracic surgical practice.

In summary, although immunotherapy holds great promise for improving surgical outcomes in NSCLC, addressing financial barriers, educational deficits, and infrastructure limitations is crucial for its successful integration. Policymakers, professional societies, and healthcare providers must collaborate to develop tailored solutions that promote equitable access and optimize the benefits of immunotherapy in thoracic surgery.

## CONCLUSION

Immunotherapy is widely accepted among thoracic surgeons, but barriers such as educational gaps, financial constraints, and access disparities remain. Addressing these challenges is crucial for optimizing the integration of immunotherapy into surgical practice. Future efforts should focus on improving training programs and promoting global access to immunotherapy to ensure equitable implementation.

## INSTITUTIONAL REVIEW BOARD STATEMENT

Ethical review and approval were waived for this study due to its nature as a voluntary and anonymous survey involving healthcare professionals, without the collection of sensitive personal data or any medical intervention. According to institutional and international ethical guidelines, studies that do not involve patients, biological material, or confidential patient information do not require formal ethical approval.

## Data Availability

The datasets generated and/or analyzed during the current study are available from the corresponding author upon reasonable request.
